# Elliptic Flowers: New Types of Dynamics to Study Classical and Quantum Chaos

**DOI:** 10.3390/e24091223

**Published:** 2022-09-01

**Authors:** Hassan Attarchi, Leonid A. Bunimovich

**Affiliations:** 1 Department of Mathematics, University of California Riverside, Riverside, CA 92521, USA; 2 School of Mathematics, Georgia Institute of Technology, Atlanta, GA 30332, USA

**Keywords:** elliptic flowers billiards, chaotic and non-chaotic core, chaotic and non-chaotic tracks

## Abstract

We construct examples of billiards where two chaotic flows are moving in opposite directions around a non-chaotic core or vice versa; the dynamics in the core are chaotic but flows that are moving in opposite directions around it are non-chaotic. These examples belong to a new class of dynamical systems called elliptic flowers billiards. Such systems demonstrate a variety of new behaviors which have never been observed or predicted to exist. Elliptic flowers billiards, where a chaotic (non-chaotic) core coexists with the same (chaotic/non-chaotic) type of dynamics in flows were recently constructed. Therefore, all four possible types of coexisting dynamics in the core and tracks are detected. However, it is just the beginning of studies of elliptic flowers billiards, which have already extended the imagination of what may happen in phase spaces of nonlinear systems. We outline some further directions of investigation of elliptic flowers billiards, which may bring new insights into our understanding of classical and quantum dynamics in nonlinear systems.

## 1. Introduction

The chaos theory, as well as nonlinear dynamics as a whole, was essentially built from some “simple” basic examples. The simplicity of these examples is often questionable, especially from the point of mathematicians, who still failed to investigate a fundamental Chirikov standard map, as well as some other basic examples of chaotic systems. That is why it is important, especially for physicists, to have a decent collection of completely (rigorously) studied examples that demonstrate the enormous richness of nonlinear dynamics.

Recently found elliptic flowers billiards [1] provide yet another type of behavior that not only had not been observed but even never imagined before. Indeed, there are essentially very (extremely) few nontrivial well-understood, and rigorously investigated examples with the coexistence of several ergodic components in the phase space of a system under study. In elliptic flowers (EF) billiards, there are three ergodic components. One of them is called a core. The two other ergodic components consist of orbits that move around the core clockwise and counter-clockwise, respectively. Therefore, EF billiards have much richer and more interesting dynamics than track billiards [2], where the entire phase space consists of two tracks, and orbits move in opposite directions to each other.

A large class of EF billiards was constructed in [1], and it was shown that in some simple sub-classes of EF, the dynamics in the core and tracks are chaotic, or the dynamics in all these three parts of the phase space can also be non-chaotic. By chaotic, we mean dynamics with an exponential divergence of nearby orbits and mixing (i.e., decay of correlations).

In the present paper, we give examples of EF billiards with opposite types of dynamics in tracks and the core. In other words, in these examples, a chaotic core coexists with non-chaotic tracks and vice versa. Therefore, all four possible types of coexistence of dynamics in the core and tracks are possible.

It is worth mentioning that EF billiards can have even more rich behavior. Particularly, EF billiards demonstrate a complicated evolution of the wavefronts (for example, see the videos by Nils Berglund on YouTube (https://www.youtube.com/c/nilsberglund) 7 June, 9 June, 11 June and 25 June 2021) and certainly could be used for studies of various types of the evolution of such waves (and wavefronts) with time in a lesson of dynamics. Another natural direction of studies is the analysis of global bifurcations that occur under a change of parameters as well as of local bifurcations of wavefronts. In addition, it turned out that track billiards are useful for some problems in quantum chaos, for example, in the studies of the Schnirelman’s peak [3]. Further, a class of billiards that is more general and abstract than tracks was considered in [4,5] to study quantum ergodicity. Thus, it seems reasonable to assume that EF billiards will help to understand something new about quantum chaos, especially in relation to the Berry–Robnik theory.

## 2. Construction/Definition of Elliptic Flowers Billiards

The general construction of a (multilayered) elliptic flower billiard (EF-billiard) starts with a choice of any convex (base) polygon *A* on the Euclidean plane. Let $A_{1},\;A_{2},\;\ldots ,\;A_{n}$ be the vertices of *A*. The first elliptic layer over *A* is formed by all arcs of ellipses, which do not intersect *A* and have focuses at the points $A_{i}$ and $A_{i+1}$, where *i* varies between 1 and *n*, and i+1 is taken as the modulus *n*. In other words, the first layer of ellipses over polygon *A* consists of all ellipses with focuses at the ends of one and the same side of *A*. The second layer of ellipses over *A* is a union of all ellipses that have focuses at the points $A_{i}$ and $A_{i+2}$, where again i+2 is taken modn. Thus the second layer of ellipses over polygon *A* consists of all ellipses with focuses at the ends of the small diagonals of *A*, which connect the ends of two neighboring sides of *A*.

Analogously, the *m*th layer of ellipses, where $m<\frac{n+1}{2}$, over a given polygon is defined in the same way, where instead of (i+2)modn, one should take (i+m)modn. Therefore, all ellipses in all layers have a focus at some vertex of *A*. It is easy to see that a convex polygon with *n* vertices has $\frac{n}{2}$ layers if *n* is an even number, and $\frac{n-1}{2}$ layers if *n* is odd. If a base polygon *A* is a triangle, then the sides also play the role of small diagonals. Therefore, in this case, there is only one layer.

**Definition** **1** ([1]). *A simply connected billiard table Q(A) is called an elliptic flower over a convex base polygon A if the following conditions are satisfied:*

*1*. *A is a subset of the billiard table, which does not intersect the boundary ∂Q(A).*
*2*. *the boundary ∂Q(A) consists of pieces of ellipses that belong to the layers of ellipses over A.*

We say that an invariant subset of an EF billiard is a track if it consists of all orbits that move clockwise or counter-clockwise in a billiard table of the corresponding EF billiard. Clearly, if there is a clockwise track, then also exists a counter-clockwise track. The orbits in tracks move around some subset Co of the EF billiard table, and they never intersect it. A core consists of all orbits that intersect Co between any two consecutive reflections off the boundary of an EF billiard table. Clearly, if *A* is a triangle or a rectangle, then the lines passing through the sides of *A* never intersect outside this polygon. It is easy to see [1] that interesting dynamics of elliptic flowers billiards appear when they are built over convex polygons with at least five sides. The following definition singles out a special subclass of EF billiards.

**Definition** **2** ([1]). *Consider a base polygon A with the vertices $A_{1},\;A_{2},\cdots ,\;A_{n}$. A simply connected billiard table Q is called a structural elliptic flower (SEF) over A if all regular components of the boundary ∂Q are the arcs of the ellipses from the layers over A, which are not allowed to cross the lines passing through the sides of A, but they may have their endpoints on these lines.*

Clearly, not all boundary components of general EF billiards may have their endpoints on the straight lines passing through the sides of *A*. However, here for the sake of simplicity, we are considering much more narrow classes of EF, for which, particularly, the construction of elliptic flowers over a base polygon *A* becomes trivial. A natural difficulty in the construction of general-type elliptic flowers is how to finish (close) the boundary by the last elliptic arc when all other regular components of the boundary were already built. Such an ellipse, which passes through two fixed points of the boundary, and has focuses at some vertices of *A*, may just not exist.

Although we expect that general elliptic flowers billiards may well demonstrate a large variety of dynamics, our concern here is to present the (hopefully) simplest examples of EF billiards with interesting nontrivial dynamics, which, to the best of our knowledge, have never been seen before.

We will start with considering an even simpler class of EF billiards, which are called the special one-layer (SOL) elliptic flowers [1]. It was proven in [1] that in some classes of SOL EF billiards a chaotic core coexists with chaotic tracks, and in some other classes of such billiards, a non-chaotic core coexists with non-chaotic tracks.

To construct EF billiards, where the coexisting core and tracks demonstrate not similar but different types of dynamics, which is a goal of the present paper, we need to move beyond SOL elliptic flowers billiards. However, our examples will be obtained as some kinds of perturbations of the SOL EF billiards.

**Figure 1:**
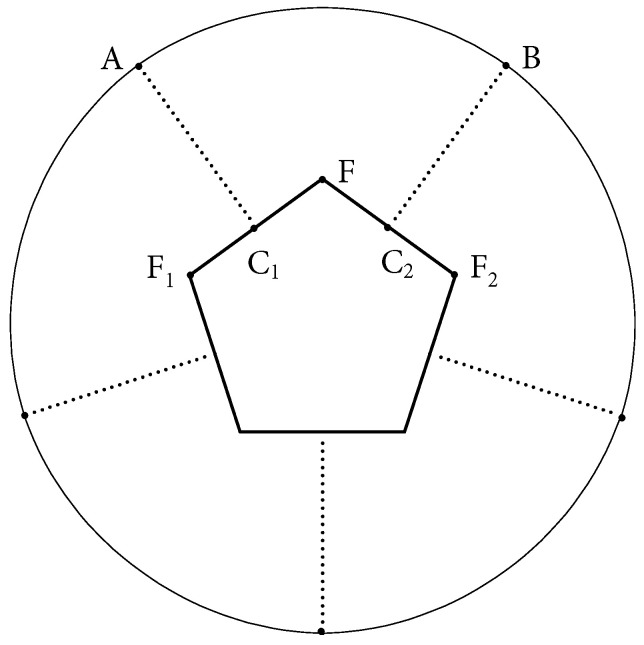
Demonstration of an SOL EF on a pentagon. The boundary component AB is a piece of an ellipse with focuses at $F_{1}$ and $F_{2}$, where they are endpoints of a small diagonal of the pentagon. Note that, in the SOL EF structure, line segments $C_{1}A$ and $C_{2}B$ are perpendicular bisectors of the sides $F_{1}F$ and $FF_{2}$, respectively.

**Construction of special one-layer elliptic flowers:** Let *A* be a regular convex polygon with *n* vertices. Consider the straight semi-lines, which have the ends at the centers of the sides of *A*, are orthogonal to the corresponding sides and do not intersect *A*. Then a special one-layer elliptic flower over *A* is a billiard table with *n* identical boundary components, which are the arcs of ellipses with the focuses located at the ends of the small diagonals of *A*. Moreover, the endpoints of each elliptic arc lie on two semi-lines orthogonal to a pair of neighboring sides of *A*, i.e., the sides of *A* that have a common vertex (See Figure 1).

## 3. Non-Chaotic Core and Chaotic Tracks

In this section, we construct examples of elliptic flowers billiards, such that the dynamics in the core are non-chaotic while the dynamics in the tracks are chaotic.

Consider a special one-layer (SOL) elliptic flower built over a regular hexagon. Let *a*, *b*, and *c* denote the lengths of the semi-major axis, semi-minor axis, and linear eccentricity of the ellipses, containing the boundary components, respectively. In this EF structure, if we assume that the lengths of the hexagon’s sides equal 2, then $c=\sqrt{3}$. If b=3, then the centers of all maximal osculating circles of the boundary components coincide with the center of the hexagon. Recall that an osculating circle to a curve γ at a point *x* is the circle tangent to γ at *x* and, the radius of this circle is 1/|κ(x)|, where κ(x) is the curvature of γ at point *x*. Clearly, the maximal osculating circles of an ellipse are tangent to the boundary at the endpoints of the minor axis. Let *b* be (slightly) greater than 3. This assumption guarantees that the core and tracks are chaotic (see Theorem 3.9 in [1]).

**Figure 2:**
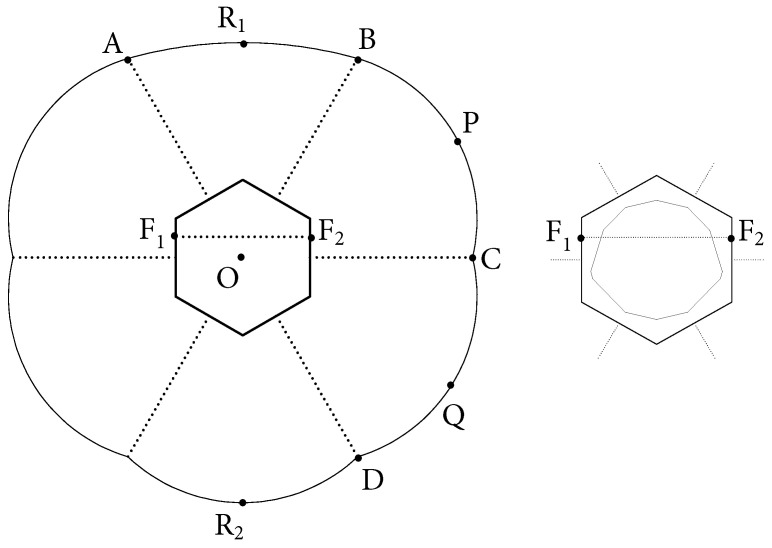
Deformed SOL EF on a hexagon (**left**), and its core (**right**).

To get a non-chaotic core, we change one of the boundary components of the EF billiard by moving its major axis toward the center *O* of the hexagon. In Figure 2, component AB has focal points $F_{1}$ and $F_{2}$ instead of endpoints of its corresponding short diagonal in the usual SOL EF structure.

Let $a_{1}$ and $b_{1}$ be the lengths of a semi-major and a semi-minor axis of AB, respectively. Note that the linear eccentricity of AB remains equal to $c=\sqrt{3}$. Now, we fix parameter *b*, and check for which range of parameter $b_{1}$ the 2-periodic orbit $R_{1}R_{2}$ is stable (Figure 2), while all periodic orbits in the tracks are unstable.

Consider a Cartesian coordinate with the center at *O*. Then

$$F_{1}=\left(-\sqrt{3},y_{0}\right),\;and\;\;F_{2}=\left(\sqrt{3},y_{0}\right),$$

where $0\leq y_{0}<1$. By fixing b=4, we also fix the points *A* and *B* in our coordinate system. Therefore, we can express $a_{1}$ (also, $b_{1}$) as a function of $y_{0}$, where

(1) $$2a_{1}=|AF_{1}|+|AF_{2}|.$$

Under these assumptions, the distance between $R_{1}$ and $R_{2}$ is given by

(2) $$|R_{1}R_{2}|=\left(b+1\right)+\left(b_{1}+y_{0}\right)=5+b_{1}+y_{0}.$$

Let $r_{1}$ and $r_{2}$ be the radii of osculating circles at $R_{1}$ and $R_{2}$, respectively. Then,

(3) $$r_{1}=\frac{a_{1}^{2}}{b_{1}}=b_{1}+\frac{c^{2}}{b_{1}}=b_{1}+\frac{3}{b_{1}},\;r_{2}=\frac{a^{2}}{b}=b+\frac{c^{2}}{b}=4.75\;.$$

Let $y_{0}=1$ in (1). Then we have $a_{1}=a$. Further, it is a direct consequence of the geometry of the deformed SOL EF that $a_{1}>a$, when $0\leq y_{0}<1$. Therefore, $b_{1}>b=4$ if $0\leq y_{0}<1$. Hence, (2) and (3) imply that

(4) $$|R_{1}R_{2}|>r_{1},\;\left|R_{1}R_{2}\right|>r_{2}.$$

The Jacobians of the billiard maps $T_{R_{1}R_{2}}$ and $T_{R_{2}R_{1}}$, corresponding to the trajectories from $R_{1}$ to $R_{2}$ and vice versa, are equal to [6]:

$$dT_{R_{1}R_{2}}=\left[\begin{array}{cc} |R_{1}R_{2}|/r_{1}-1 & -|R_{1}R_{2}|\\ -|R_{1}R_{2}|/\left(r_{1}r_{2}\right)+1/r_{1}+1/r_{2} & |R_{1}R_{2}|/r_{2}-1 \end{array}\right],$$

and

$$dT_{R_{2}R_{1}}=\left[\begin{array}{cc} |R_{1}R_{2}|/r_{2}-1 & -|R_{1}R_{2}|\\ -|R_{1}R_{2}|/\left(r_{1}r_{2}\right)+1/r_{1}+1/r_{2} & |R_{1}R_{2}|/r_{1}-1 \end{array}\right].$$

Therefore,

$$trace\left(dT_{R_{1}R_{2}}dT_{R_{2}R_{1}}\right)=2-4\left(\frac{|R_{1}R_{2}|}{r_{1}}+\frac{|R_{1}R_{2}|}{r_{2}}-\frac{|R_{1}R_{2}{|}^{2}}{r_{1}r_{2}}\right).$$

For all values of $y_{0}\in \left[0,\;0.414569\right)$, we obtain

(5) $$|trace(dT_{R_{1}R_{2}}dT_{R_{2}R_{1}})|<2,$$

and

(6) $$r_{1}+r_{2}>\left|R_{1}R_{2}\right|.$$

Inequality (5) shows that the 2-periodic orbit between $R_{1}$ and $R_{2}$ is linearly stable. In order to demonstrate that this 2-periodic orbit is, in fact, elliptic (nonlinearly stable), we must check that the first Birkhoff coefficient is nonzero.

In the ellipse E={(acosθ,bsinθ)|0≤θ<2π}, the radius of curvature r(θ) can be expressed as

$$r\left(\theta \right)=\frac{{\left(\sqrt{a^{2}\sin^{2}\theta +b^{2}\cos^{2}\theta }\right)}^{3}}{ab}.$$

Let *s* be the arc length parameter of the ellipse *E*. Then

(7) $$\frac{dr}{ds}\left(\theta \right)=\frac{3(a^{2}-b^{2})\sin\theta \cos\theta }{ab},$$

and

(8) $$\frac{d^{2}r}{ds^{2}}\left(\theta \right)=\frac{3\left(a^{2}-b^{2}\right)\left(\cos^{2}\theta -\sin^{2}\theta \right)}{ab\sqrt{a^{2}\sin^{2}\theta +b^{2}\cos^{2}\theta }}.$$

Let $r_{1}^{\prime }$ and ${r_{1}}^{\prime \prime }$ (also, $r_{2}^{\prime }$ and ${r_{2}}^{\prime \prime }$) be the first and second derivatives of the radius of curvature at the point $R_{1}$ (also, $R_{2}$), respectively. From Equations (7) and (8), we have

(9) $$r_{1}^{\prime }=r_{2}^{\prime }=0,\;{r_{1}}^{\prime \prime }=\frac{-3(a_{1}^{2}-b_{1}^{2})}{a_{1}^{2}b_{1}},\;{r_{2}}^{\prime \prime }=\frac{-3(a^{2}-b^{2})}{a^{2}b}.$$

Denote by $\tau_{1}$ the first Birkhoff coefficient (see [7] for the explicit formula for $\tau_{1}$ corresponding to an elliptic 2-periodic orbit) of the 2-periodic orbit between $R_{1}$ and $R_{2}$. Then, by making use of (3) and (9), we obtain that

(10) $$\tau_{1}=-\frac{1}{8}\frac{r_{1}+r_{2}}{r_{1}r_{2}}-\frac{1}{8}\frac{|R_{1}R_{2}|}{|R_{1}R_{2}|-r_{1}-r_{2}}\left(\frac{|R_{1}R_{2}|-r_{1}}{|R_{1}R_{2}|-r_{2}}{r_{2}}^{\prime \prime }+\frac{|R_{1}R_{2}|-r_{2}}{|R_{1}R_{2}|-r_{1}}{r_{1}}^{\prime \prime }\right).$$

It follows from (3), (4), (6), and (9) that $\tau_{1}$ is nonzero because both terms on the right-hand side of (10) are strictly negative. Therefore, the 2-periodic orbit $R_{1}R_{2}$ is elliptic (nonlinearly stable). Hence, the global dynamics in the core are non-chaotic if $y_{0}\in \left[0,0.414569\right)$ because an elliptic island with a positive measure exists. One should expect, though, that there are some subsets of the core where dynamics are chaotic.

We will check for what subrange of parameter $y_{0}$, with the same choice of other parameters, the dynamics in tracks is chaotic. First, we check that all boundary components are absolutely focusing curves. The boundary consists of six pieces of ellipses. Five of them have the parameters b=4, $c=\sqrt{3}$, and $a=\sqrt{19}$. Let the projections of these five pieces to the corresponding major axis of the ellipses have the length *L*. Then

$$L<2\left(b+1\right)\tan\frac{\pi }{6}=\frac{10}{\sqrt{3}}.$$

It implies that

(11) $$L<\frac{10}{\sqrt{3}}<\sqrt{38}=a\sqrt{2}.$$

It was proven in [8] that a piece of an ellipse (symmetric with respect to its minor axis) is absolutely focusing if its projection to the major axis has a length less than $a\sqrt{2}$. Thus, (11) shows that the similar five pieces of ellipses are absolutely focusing. The projection of the boundary component AB to the major axis of the corresponding ellipse also has the length *L*. Indeed, it immediately follows from our construction, since *A* and *B* are endpoints of the boundary components in a SOL EF. Because $a_{1}>\sqrt{19}=a$, the boundary component AB is also absolutely focusing (i.e., $L<a_{1}\sqrt{2}$). Thus, all six boundary components are absolutely focusing in this deformed SOL EF. Moreover, the angles between adjacent (focusing) boundary components are greater than π. Because of these facts, we only need to show that the boundary components are sufficiently far apart from each other, which will imply that the dynamics in the tracks are chaotic.

On the other hand, if $y_{0}\in \left[0.40357,0.414569\right)$, then

(12) $$r_{1}\cos\phi_{1}+r_{2}\cos\phi_{2}<\left|R_{1}P\right|,$$

and

(13) $$r_{1}\cos\phi_{3}+r_{2}\cos\phi_{4}<\left|R_{1}Q\right|,$$

where *P* and *Q* are points at the end of minor axes of components BC and CD, respectively, and

- $\phi_{1}$: the angle between the normal line at $R_{1}$ and the line segment $R_{1}P$,
- $\phi_{2}$: the angle between the normal line at *P* and the line segment $R_{1}P$,
- $\phi_{3}$: the angle between the normal line at $R_{1}$ and the line segment $R_{1}Q$,
- $\phi_{4}$: the angle between the normal line at *Q* and the line segment $R_{1}Q$.

Inequalities (12) and (13) imply that the tangent circles of radius $\frac{r_{1}}{2}$ and $\frac{r_{2}}{2}$ at $R_{1}$ and *P* (or *Q*), respectively, do not cover the entire segment $R_{1}P$ (or $R_{1}Q$). Therefore, the boundary components are sufficiently far apart from each other to have hyperbolicity (generated by the defocusing mechanism) of trajectories traveling between them [8,9,10]. We also showed before that boundary components are absolutely focusing. Therefore, it follows from the general theory of billiards [6,9,10] that the dynamics in tracks are hyperbolic and chaotic thanks to the mechanism of defocusing.

## 4. Chaotic Core and Non-Chaotic Tracks

In this section, we construct examples of elliptical flowers with a chaotic core and non-chaotic tracks.

Consider a SOL EF billiard table built over a regular pentagon. Let *a*, *b*, and *c* denote the semi-major axis, semi-minor axis, and linear eccentricity of the elliptical components of the boundary, respectively. Let the lengths of the sides of the pentagon equal 2. Then $c=\cos\frac{\pi }{5}$. Moreover, if we choose

$$b=c\tan\frac{2\pi }{5}=\cos\frac{\pi }{5}\tan\frac{2\pi }{5}≃2.489898,$$

then the centers of the osculating circles with the maximum radius of all regular components of the boundary coincide with the center of the pentagon. Choose now *b* to be slightly smaller than $c\tan\frac{2\pi }{5}≃2.489898$. More precisely, we assume that

(14) 2.484<b<2.489898.

**Figure 3:**
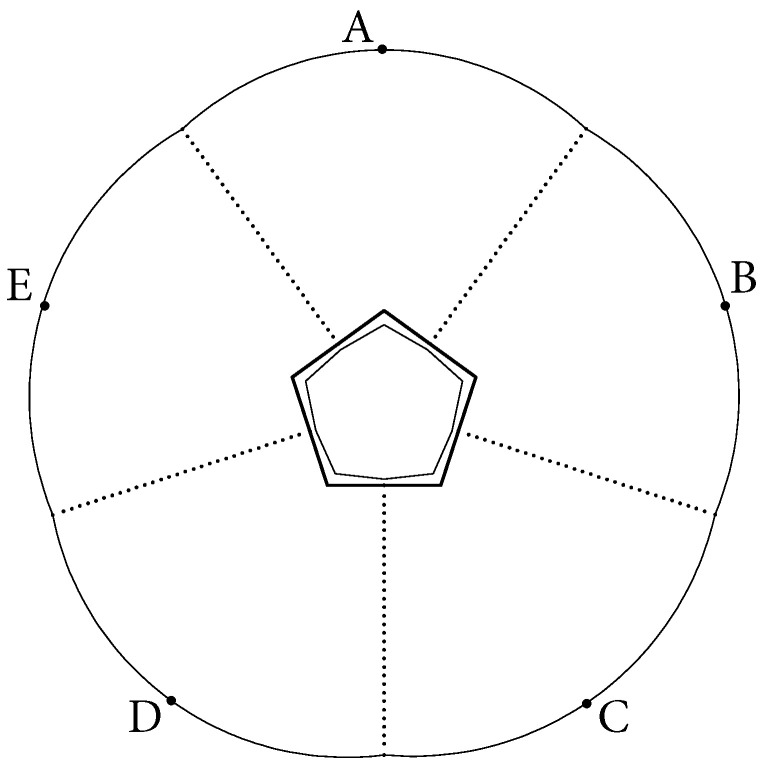
A SOL EF on a pentagon, and its actual core is a decagon.

Under this assumption, we will show that the (pentagon shape) 5-periodic orbit ABCDE (Figure 3) is a linearly stable orbit in a track. Clearly, then the 5-periodic orbit, which traces the same points in the opposite direction, is also linearly stable. We have

$$\tau :=\left|AB\right|=\left|BC\right|=\left|CD\right|=\left|DE\right|=\left|EA\right|=2\left(b+\cos\frac{\pi }{5}\tan\frac{\pi }{10}\right)\cos\frac{3\pi }{10}.$$

Moreover, the angles of reflection φ and the curvatures κ of the boundary at points *A*, *B*, *C*, *D*, and *E* are equal to $\frac{3\pi }{10}$ and $\frac{b}{a^{2}}$, respectively.

The jacobians of the billiard map corresponding to consecutive reflections along this periodic orbit are equal to

$$dT=\left[\begin{array}{cc} X & Y\\ Z & X \end{array}\right]=\frac{-1}{\cos\varphi }\left[\begin{array}{cc} -\tau \kappa +\cos\varphi & \tau \\ \tau \kappa^{2}-2\kappa \cos\varphi & -\tau \kappa +\cos\varphi \end{array}\right],$$

and

$$trace\left({\left(dT\right)}^{5}\right)=2X^{5}+10XY^{2}Z^{2}+20X^{3}YZ.$$

Under the assumption (14), we obtain that

$$|trace({\left(dT\right)}^{5})|<2.$$

Hence, if *b* is slightly smaller than $c\tan\frac{2\pi }{5}$, then there are linearly stable periodic orbits in the tracks. Therefore, the dynamics in the tracks are not globally chaotic, although generically, some subset(s) with chaotic dynamics (chaotic seas) should be present there. However, at this stage of analysis of EF billiards, we are only concerned with global chaos (or with the absence of global chaos) in the tracks and in the core.

**Figure 4:**
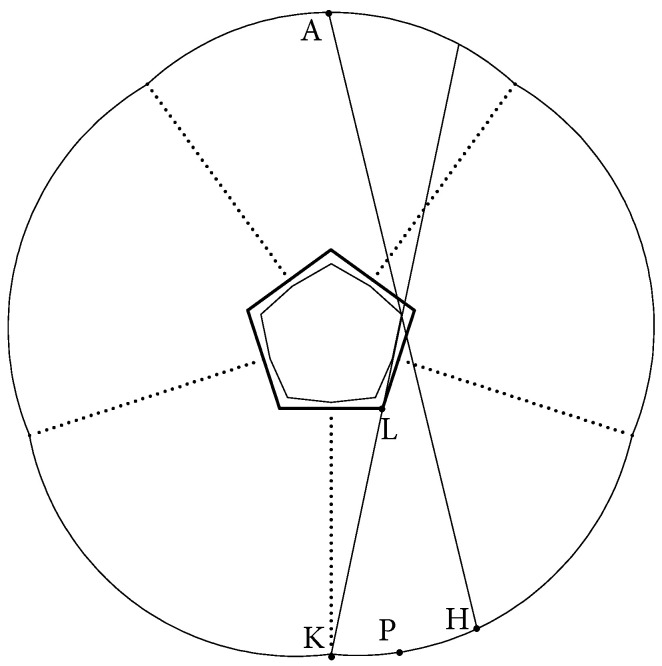
Continuation of all segments similar to KL cuts out from the pentagon a smaller polygon, which becomes a core.

We will show now that the dynamics in the core (which is a decagon in this EF billiard) are chaotic. The way how a decagon core appears from a EF billiard table, with a pentagon as a base polygon, is demonstrated in Figure 4 (this process is described in detail in [1]). The reason is that the lines connecting the ends of the boundary components to the corner points of the pentagon (as point *K* is connected to point *L* in Figure 4) cut out pieces of the base pentagon. The remaining subset of the pentagon, which forms a core of the EF billiard, is a decagon.

Let point *A*, see Figure 4, be the endpoint of the minor axis of a boundary component of this billiard table. All straight lines, which start at point *A* and pass through the core (decagon) form a cone with vertex *A*, where AH belongs to the boundary of this cone (Figure 4). A simple computation for any point such as *P* on the boundary between *H* and *K* shows that

(15) $$r_{A}\cos\phi_{A}+r_{P}\cos\phi_{P}<\left|AP\right|,$$

where $r_{A}$ and $r_{P}$ are radii of curvature at the points *A* and *P*, respectively, and

- $\phi_{A}$: the angle between the normal line at *A* and the line segment AP,
- $\phi_{P}$: the angle between the normal line at *P* and the line segment AP.

Inequality (15) implies that the (osculating) tangent circles with the radii $\frac{r_{A}}{2}$ and $\frac{r_{P}}{2}$ at *A* and *P*, respectively, do not cover segment AP. This observation, together with the fact that trajectories passing through the core cannot have two consecutive reflections off one and the same boundary component, prove that all orbits in the core are hyperbolic (due to the defocusing mechanism). Therefore, the dynamics in the core are hyperbolic and chaotic.

## 5. Concluding Remarks

This paper, together with [1], shows that all four possible types of coexistence of dynamics in the core and in tracks are realized in the elliptic flowers billiards. Therefore, dynamics could be chaotic in the core and tracks, it could be chaotic in the core and non-chaotic in tracks, it could be non-chaotic in the core and chaotic in tracks, and, finally, the dynamics could be non-chaotic both in the core and in tracks.

However, these results just scratch the surface of this new area. General multilayered elliptic flowers billiards, especially the ones built over non-regular polygons, would likely demonstrate a more rich variety of behaviors. The analysis of standard (without a core) track billiards allowed some results in classical and quantum chaos to be obtained [3,11]. There is little doubt that studies of general EF billiards will bring something new, interesting, and surprising. A great challenge is to construct such billiards (or other dynamical systems similar to EF billiards dynamics) in dimensions greater than two. It has actually been performed for track billiards [2]. However, the construction of high-dimensional elliptic flowers seems to be a real challenge.

## Data Availability

Not applicable.
